# Phosphate-activated geopolymers: advantages and application

**DOI:** 10.1039/d3ra05131e

**Published:** 2023-10-16

**Authors:** Kristina Goryunova, Yunis Gahramanli, Vida Muradkhanli, Parviz Nadirov

**Affiliations:** a Department of “Technology of Inorganic Substances and Chemistry” Azerbaijan State Oil and Industry University Baku Azerbaijan kristina.goryunova@hotmail.com y.gahramanli@asoiu.edu.az

## Abstract

Silica-aluminophosphate (SAP) geopolymers are a novel type of green mortar made from aluminosilicate precursors and phosphoric acid (PA), and they are attracting the interest of researchers due to their extraordinary and distinctive capabilities. According to current research, SAP geopolymers have great mechanical properties, high heat and fire resistance, and outstanding sorption activity. Because of their properties, they have a wide range of applications, including novel insulating, construction, coating, and wastewater treatment materials. This paper focuses on the most recent advances in SAP geopolymer research. Furthermore, this work indicates novel applications for SAP geopolymers, which might serve as guidance for future research activity of scientists.

## Abstract 2

This paper focuses on the most recent advances in phosphate-activated geopolymer research, and indicates novel applications for phosphate-activated geopolymer.

## Graphical abstract

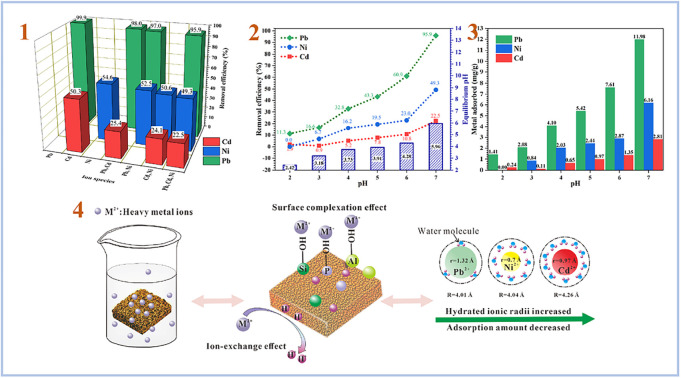

## 1. Introduction

Geopolymers are three-dimensional inorganic polymeric materials constituted of cross-linked tetrahedral units [AlO_4_] and [SiO_4_] and alkali metal cations. Geopolymers, a novel type of inorganic nonmetallic material with remarkable performance features, are garnering increasing interest from material scientists and technologists after more than 40 years of research, particularly in the last 20 years.

Prof. Joseph Davidovits, a French scientist and engineer, established the name “geopolymer” in the 1970s to describe a family of solid materials formed by the interaction of an aluminosilicate powder with an alkaline solution.^1,2^ Following a series of fires in Europe, these materials were initially developed as a fire-resistant alternative to organic thermosetting polymers, and products based on this initial work have since found application as coatings for fire protection for cruise ships,^3,4^ as a resin in high-temperature carbon-fire composites,^5,6^ as a heat-resistant adhesive,^6,7^ as a monolithic refractory,^8,9^ and a variety of other niche applications.

Geopolymers are a subgroup of the wider class of alkali-activated binders,^10^ which also includes materials generated by alkali-, silicate-, carbonate-, or sulfate-activation of metallurgical slags, yielding mostly calcium silicate hydrate. A geopolymer is distinguished by the presence of an alkali aluminosilicate gel in the binding phase, with aluminum and silicon connected in a three-dimensional tetrahedral gel structure that is particularly resistant to dissolution in water.^11,12^

**Fig. 1:**
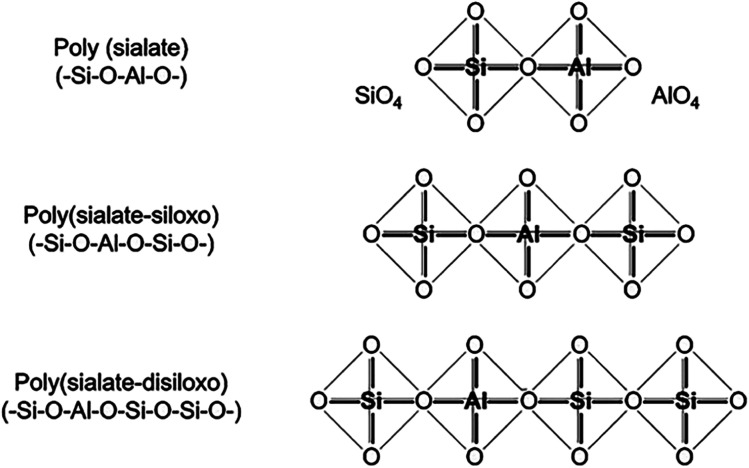
Structural unit model of geopolymer with different Si/Al ratios, Davidovits *et al.*, *J. Therm. Anal.*, 2008.^2^

Davidovits established the “sialate” terminology to designate aluminosilicate formations more than 40 years ago.^13^ Si–O–Al coupling was dubbed a sialate bond, and Si–O–Si a siloxo bond. This allowed to describe the composition of geopolymers based on their Si/Al ratio, with a ratio of 1.0 indicating a poly(sialate), 2.0 indicating a poly(sialate-siloxo), and 3.0 indicating a poly(sialate-disiloxo) (Fig. 1).

**Fig. 2:**
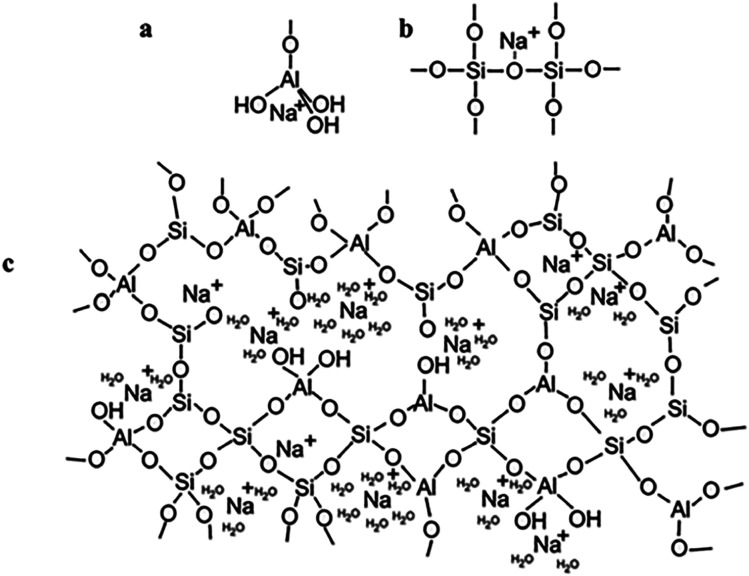
(a) Proposed model for the location of the charge-balancing Na^+^ cation which assumes the form of a semi attached Na aluminate species; (b) proposed model for the location of the charge-balancing Na^+^ cation which assumes the form of a modified bridging network; (c) proposed schematic model for alkali-activated geopolymer structure based on an original model. This figure has been adapted/reproduced from ref. 14 with permission from Springer Nature under the license number 5632330146683, copyright 2023.

To begin with, geopolymers are a complicated class of materials. The most common way to make geopolymers is to mix an alkaline solution with a reactive aluminosilicate powder, such as metakaolin or fly ash. This leads to the creation of the geopolymeric gel binder phase, a disordered alkali aluminosilicate gel phase. This phase comprises uncreated solid precursor particles, while the pore network of the gel contains the water needed to combine the precursors, which was provided *via* an alkaline “activating solution.” The gel's fundamental framework is a highly connected three-dimensional network of aluminate and silicate tetrahedra, with the negative charge due to Al^3+^ in four-fold coordination localized on one or more of the bridging oxygens in each aluminate tetrahedron and balanced by the activating solution's alkali metal cations^14^ (Fig. 2).

The investigation of the area of acid activation of geopolymers, or phosphate geopolymers, has grown in prominence in recent years. Due to the change in molecular structure caused by phosphorus atoms, geopolymers activated by phosphoric or orthophosphoric acid have a variety of benefits, for instance, higher thermal stability, higher mechanical strength, and higher sorption capacity.

The economic aspect might also be a crucial signal in the manufacture of geopolymer cement. Alkaline-activators, such as sodium silicate and sodium hydroxide, are frequently used in the development of appropriate materials for civil engineering applications, such as geopolymer cements. Due to the high cost of sodium silicate, this factor has a significant impact on the final product's pricing. As a result, phosphoric acid-based geopolymers are the ideal replacement for alkaline-based geopolymers.

This study describes the primary challenges in current acid-activated geopolymers research and provides prospective applications, which will serve as a guide for future research efforts or relevant scholars.

## 2. Synthesis and classification

Geopolymers are formed from a reaction between an aluminosilicate precursor and an alkali or acid activator.

Alkali-activated geopolymers are formed in the presence of alkaline activators (such as NaOH and sodium silicate), and their chemical structure units include –Si–O–Al–O– and –Si–O–Al–O–Si–O– units.^15,16^ In phosphoric acid (PA) medium, acid-activated geopolymers are formed, and their chemical structural units consist of –Al–O–P–O–, –P–O–Si–O–P–O–, –P–O–Si–O–Al–O–P–O–, and –P–O–P–O– units.^2,17^ Alkali-activated geopolymers have garnered increasing attention in recent decades, but acid-activated geopolymers have progressed more slowly. However, in engineering applications, acid-activated geopolymers may have durability and acidity concerns. Dissolution, speciation, gelation and rearrangement, and polymerization/polycondensation are all mechanisms of these two binders.^18^ The inclusion of contaminants such as calcium, magnesium, iron, and others influences the composition and characteristics of these binders. For example, the presence of iron in alkaline media causes some Al to be replaced by Fe, resulting in ferro-aluminosilicate gel. The inclusion of Ca^2+^ in the same medium enhances the development of C–S–H forms such as tobermolite and transforms N–A–S–H gel to N–C–A–S–H.^19^ The presence of iron in an acidic medium causes a rapid reaction between phosphoric acid and magnetite (Fe_3_O_4_) to generate the amorphous gels Fe(H_2_PO_4_)_2_, Fe(H_2_PO_4_)_3_, FeHPO_4_, and Fe_2_(HPO_4_)_3_.^20^ Given the differences, the adsorption rates, adsorption capacities, and adsorption methods onto silica-aluminophosphate (SAP) and alkali-aluminosilicate (AAS) geopolymers must be different and cannot be predicted in advance.

### 2.1. Preparation process of geopolymers

#### 2.1.1 Raw materials

Clay minerals are an important source of aluminosilicate for the production of geopolymers.^21^ Metakaolin (MK), a calcination derivative of kaolinite, is often utilized to make geopolymers due to its excellent pozzolanic characteristics.^22,23^ With growing concern about pollution and clean manufacturing, the utilization of industrial solid waste as raw materials for geopolymers has attracted increased attention internationally in recent years. Several solid wastes containing reactive silica and alumina, such as fly ash (FA),^24^ granulated blast-furnace slag,^25,26^ biomass ash,^27^ and red mud,^28^ have been identified as promising candidate raw materials for SAP geopolymerization.

To increase pozzolanic activity, kaolinite is calcined (500–900 °C) to produce metakaolinite.^29^ Metakaolin is predicted to be soluble in these systems due to the existence of a significant amorphous aluminosilicate phase that dissolves easily in sodium hydroxide solutions.^30^

Because of their consolidation effect, tailings are frequently utilized as a raw material for concrete or cement, particularly heavy-metal-contaminated tailings.^31^ Acid-based geopolymers, in the contrary, have better bonding and consequently higher compressive strengths than alkali-based geopolymers.^32^

Zhang *et al.* (2020)^33^ synthesized geopolymer using the spent fluid catalytic-cracking (SFCC) catalyst instead of metakaolin, and the material's highest compressive strength was 41.22 MPa. Bouzón *et al.* (2014)^34^ combined the SFCC catalyst and rice husk ash to produce geopolymers. The compressive strength of geopolymer was found to be between 31 and 41 MPa. However, the authors employed alkali-activation of geopolymers in these investigations, whereas Wan *et al.* (2022)^35^ used SFCC catalyst as a raw material for the formation of phosphate geopolymers with a maximum compressive strength of 30.2 MPa.

**Fig. 3:**
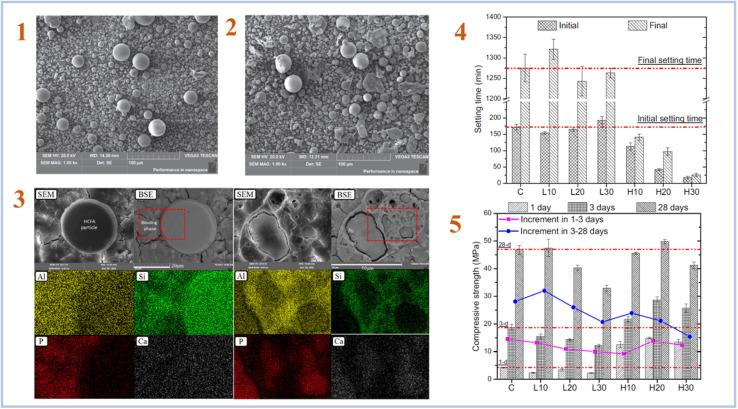
(1) SEM image of LCFA; (2) SEM image of HCFA; (3) typical backscattered electron images and elemental mapping results of the H30 geopolymer after 28 days curing; (4) initial and final setting time of the SAP geopolymers with different types and contents of CFA; (5) compressive strength results of the FA-SAP geopolymers with different types and contents of FA. This figure has been adapted/reproduced from ref. 36 with permission from Elsevier under the license number 5632320548892, copyright 2023.

Fly ash is a form of industrial solid waste made mostly of CaO, Al_2_O_3_, and SiO_2_ by coal-fired power plants. Fly ash (FA) is often classified as high-calcium fly ash (HCFA) or low-calcium fly ash (LCFA) based on its CaO level. Wang *et al.* (2020)^36^ investigated the micromorphology and geopolymerization mechanisms of HCFA and LCFA. Fig. 3 represents SEM images of HCFA and LCFA, setting time of samples, elemental mapping and compressive strength results.

Further investigations suggest that adding solid wastes such as blast furnace slag and electrolytic manganese slag may speed up the geopolymerization procedure, and the resulting geopolymer samples have good water resistance and high temperature resistance.^25,26^

Table 1 shows the various kinds and constituents of precursors employed in the synthesis of SAP geopolymers.

**Table 1:** Main components of commonly used aluminosilicate precursors^a^

Precursor		Chemical composition (wt%)									Ref.
		SiO_2_	Al_2_O_3_	CaO	Fe_2_O_3_	MgO	TiO_2_	Na_2_O	P_2_O_5_	LOI	
Natural aluminosilicate precursors	Metakaolin	41–75	22–44	0.01–0.91	0.23–7.65	0.06–0.65	0.49–4.45	0.03–4.45	0.03–0.62	0.1–2.43	37
	Kaolinite	47.69	36.48	0.08	0.69	0.11	0.36	0.07	—	13.47	29
	Volcanic ash	42	16	9.3	13.51	8.2	3.02	2.42	0.89	2.61	38
	Raw laterite	52.3	21.68	0.08	10.68	0.12	1.29	—	0.1	—	39
	Halloysite	46	37.8	0.07	0.72	0.13	0.07	—	—	14.9	32
	Clay	60.8	16.2	2.15	5.87	2.38	—	0.003	—	9.16	40
	Limestone	38.7	13.9	34.6	—	2.291	0.095	2.634	—	8.05	41
Solid wastes	Fly ash	53.63	21.71	10.80	7.96	1.17	0.86	1.20	—	0.33	42
	LCFA*	44.5	31.2	5.3	6.5	1.9	1.2	1.1	—	3.8	36
	HCFA*	38.1	26.5	16.5	8.5	1.2	1.7	0.6	—	6.5	36
	EMDR*	10.36	4.279	0.064	8.739	0.09	—	0.052	0.096	—	43
	GGBFS*	38.0	10.8	40.1	0.3	7.24	0.83	0.31	0.02	—	26
	Mine tailings	16.2	2.6	0.4	38.9	—	0.2	—	0.3	28.1	44
	SFCC*	37.63	55.29	0.39	0.58	—	—	0.15	—	—	35
	IOE*	25.17	7.22	21.6	24.45	2.81	0.38	0.26	0.2	—	45
	HMNS*	46.66	8.44	0.92	14.01	26.53	—	—	—	—	83

a LCFA*: low-calcium fly ash; HCFA*: high-calcium fly ash; EMDR*: electrolytic manganese dioxide residue; GGBFS*: ground granulated blast furnace slag; SFCC*: spent fluid catalytic cracking catalyst; IOE*: iron ore enrichment; HMNS*: high magnesium nickel slag.

The principal mineral is kaolinite, which is commonly combined with quartz, anatase, and illite. It can also be linked with goethite, hematite, gibbsite, and halloysite, depending on the geological context. Some studies looked at the effect of secondary minerals found in kaolin on the qualities of geopolymer cements hardened using sodium silicate. The effects of halloysite in kaolin on the characteristics of geopolymer materials were explored by Zhang *et al.* (2012).^46^ The presence of halloysite in the structure of kaolin increases the reactivity of the calcined product, hence increasing the compressive strength of metakaolin-based geopolymer cements, according to the author. Rüscher *et al.* (2013)^47^ investigated the impact of quartz content in raw materials on the development of compressive strength in geopolymer cements. Essaidi *et al.* (2014)^48^ looked into the role of hematite in metakaolin aluminosilicate gels. They observed that the inclusion of iron oxide affects the synthesis of geopolymer compounds, resulting in the development of consolidated materials with the lowest compressive strength values. They stated that the compressive strengths of the collected specimens ranged from 4.0 to 27.0 MPa. Tchakouté *et al.* (2017)^49^ studied the effect of gibbsite in kaolin on the characteristics of metakaolin–phosphate-based geopolymer cements. They discovered that a larger gibbsite concentration in kaolin had a detrimental effect on the characteristics of the resulting geopolymer cements.

From all the aforementioned, it can be noted that the constitution of raw materials used in the preparation process of SAP geopolymers plays a crucial role due to the complex mechanism of formation. For SAP geopolymers, the major component must be Al_2_O_3_, because the formation of tetrahedral bonds is established by the reaction between Al^3+^ and PO_4_^3−^ groups.

Different minerals contained in the starting material can also change the properties of geopolymers, which is why it is necessary to know the mineralogical content of the raw material. Regarding the studies, the best properties were observed with quartz, halloysite, illite, and anatase, whereas gibbsite and hematite have a negative effect on the mechanical properties of SAP geopolymers.

#### 2.1.2 Curing conditions

In SAP production, the curing temperature is a crucial component that greatly affects the geopolymer's properties and curing time. SAP does not solidify and harden as quickly as AAS does at room temperature because of the delayed release of silicon and aluminum in aluminosilicate in acidic media. The metakaolin (MK) and H_3_PO_4_ solution frequently takes an excessively long time (more than 48 h) to set at room temperature,^50^ so SAP samples are frequently created at higher curing temperatures. The optimal curing temperature of SAP for constant temperature curing was found to be 70 °C, and higher curing temperatures were detrimental to SAP's properties.^43^

**Fig. 4:**
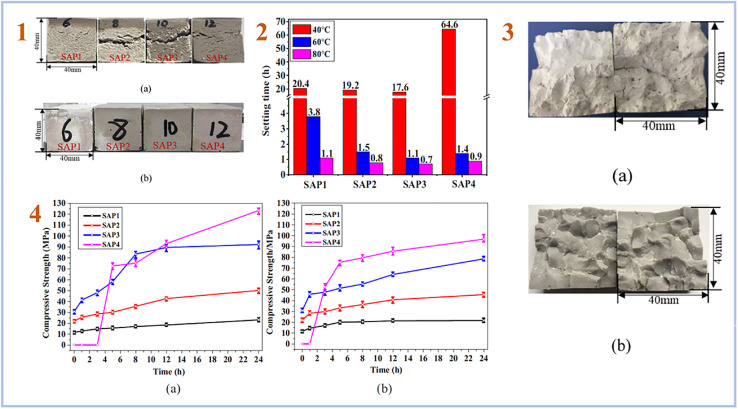
(1) appearance of the SAP samples: (a) cured at 60 °C, (b) cured by the two-stage curing method (pre-cured at 40 °C for 24 h, then cured at 60 °C/80 °C for 24 h); (2) setting time of the SAPs cured at different temperatures; (3) images of the SAP samples obtained after the two-step curing at 60 °C: (a) SAP1 with low P/Al molar ratio of 0.52, (b) SAP2-SAP4 with relatively high P/Al ratios of 0.64–0.84; (4) development of compressive strength of the SAPs in the second curing stage (a) cured at 60 °C, (b) cured at 80 °C. This figure has been adapted/reproduced from ref. 54 with permission from Elsevier under the license number 5632321036081, copyright 2023.

A multi-stage curing process is more frequently used to create SAP geopolymers, in which the mixes are typically pre-cured at high temperatures of 50–90 °C for up to 2 days, followed by a lengthy curing at room temperature.^40,51–53^ Lin *et al.* (2021)^54^ created SAP geopolymers using a two-stage curing process and found that the higher curing temperature caused significant cracking in the SAP samples due to the rapid exothermic reaction and expansive stress caused by the high internal temperature rise. The SAP samples with the highest compressive strength of 123.4 MPa were produced using a two-stage curing procedure that involved a pre-curing at 40 °C for 24 hours and a secondary curing at higher temperatures of 60 and 80 °C for another 24 hours (Fig. 4).

**Fig. 5:**
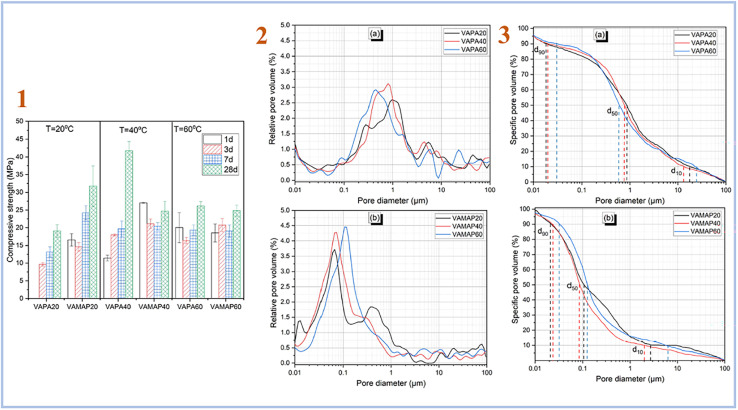
(1) Compressive strength evolution of volcanic ash phosphate geopolymer; (2) relative pore volume distribution of (a) phosphoric acid activated volcanic ash-based geopolymer binder with varying curing temperatures acid, (b) acid aluminum phosphate activated volcanic ash-based geopolymer binder with varying curing temperatures; (3) pore size distribution of (a) phosphoric acid activated volcanic ash-based geopolymer binder with varying curing temperatures, (b) acid aluminum phosphate activated volcanic ash-based geopolymer binder with varying curing temperatures, Djobo *et al.*, *RSC Adv.*, 2022.^38^

Djobo *et al.* (2022)^38^ examined the development of compressive strength of geopolymer binders cured at 20, 40, and 60 °C in another investigation. A curing temperature of 40 °C yields the greatest improvement and higher pore size (Fig. 5). When compared to diluted phosphoric acid-containing soluble aluminum, the curing temperature is the most favorable parameter for quickening the reaction kinetic. This is because heat promotes not only the dissolving of aluminum from volcanic ash, but also the solubility of other reactive components such as iron, calcium, magnesium, and, to a smaller extent, silicon.

The opposite results were obtained by Bewa *et al.* (2019).^55^ They used calcined laterite as an aluminosilicate source to explore the impact of curing temperature on the characteristics of phosphate-based geopolymers. The acid-based geopolymers were cured for 24 hours at room temperature, 40, 50, 60, 70, 80, and 90 °C. In comparison to specimens cured at 40–90 °C, whose highest compressive strength was in the range of 24.0–65 MPa, they discovered that specimens treated at room temperature had compressive strength values of 83.0 MPa or higher.

Therefore, it can be concluded that curing conditions can change the compressive strength of SAP geopolymers obtained from different starting materials. The best way to prepare SAP geopolymers with desired mechanical properties is to use a two-stage curing method, comprising low-temperature treatment at the first stage (40–50 °C) for 24–48 hours, and high-temperature treatment at the second stage (60–80 °C) for 24–48 hours.

#### 2.1.3 Concentration of activating solution

The concentration of the activating solution, such as phosphoric acid (PA), plays an important role in the synthesis of SAP geopolymers. SAP compressive strengths are generally modest after activation with low concentration PA. This was due to the polymerization reaction necessitating a greater acid dosage and curing temperature in order to achieve a high rate of Si and Al dissolving from precursor particles, which could subsequently polymerize into a three-dimensional network structure as the load bearing structure.^56^

**Fig. 6:**
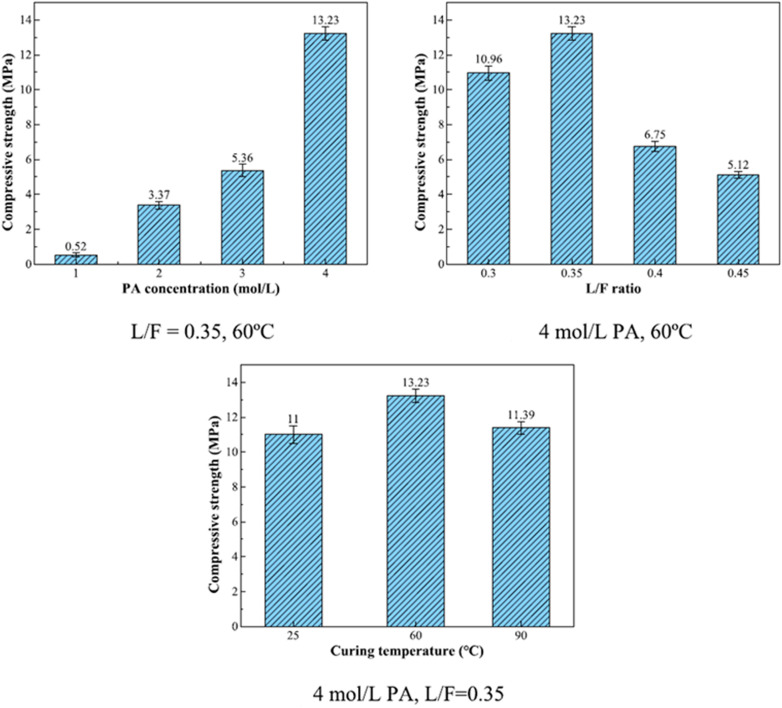
Compressive strength of SAP (L/F – liquid to fly ash ratio; PA – phosphoric acid). This figure has been adapted/reproduced from ref. 61 with permission from Elsevier under the license number 5630021114812, copyright 2023.

The principal reaction products in phosphate-based geopolymer, according to Wang *et al.* (2017),^57^ were an amorphous structure of SiO_2_·Al_2_O_3_·P_2_O_5_·*n*H_2_O and a crystalline phase, aluminum hydrogen phosphate (AlH_3_(PO_4_)·3H_2_O), which resulted in improved strength. Using 6.8 mol L^−1^ PA-activated FA, Pu *et al.* created SAP with compressive strengths ranging from 3 to 21 MPa.^58^ Mahyar *et al.* (2015),^59^ on the other hand, used 8.7 mol L^−1^ PA-activated FA to generate SAP with compressive strengths ranging from 15 to 22 MPa. As a result, increasing the concentration of PA does not always improve the mechanical characteristics of SAP. Furthermore, high PA concentrations generated significant amounts of heat during the polymerization procedure, reducing the setting of SAP, which is harmful to engineering applications and increases costs.^56,60^ In recent study, He *et al.* (2023)^61^ prepared SAP with FA and acid solutions with concentrations of 1, 2, 3, 4 mol L^−1^. They conclude that compressive strength of samples increased by 24.44% when the PA concentration was increased from 1 to 4 mol L^−1^ (Fig. 6).

**Fig. 7:**
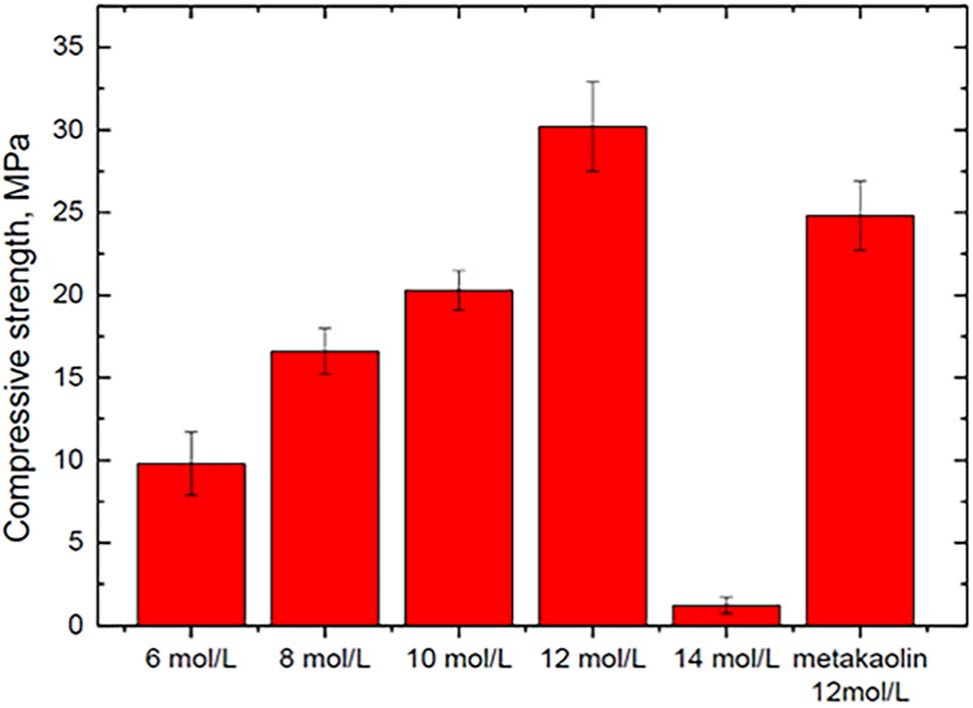
Compressive strengths of geopolymers synthesized with different concentrations of acid-activator, Wan *et al.*, Gels, 2022.^35^

Wan *et al.* (2022)^35^ investigated whether the concentration of phosphoric solutions has a significant effect on the mechanical properties of geopolymer synthesized with the SFCC catalyst using the SFCC catalyst as an aluminosilicate precursor and acid solutions with concentrations of 6, 8, 10, 12, and 14 mol L^−1^. When the acid content was between 6 and 12 mol L^−1^, a stable binder with a compressive strength ranging from 9.8 to 30.2 MPa was prepared (Fig. 7). A greater acid content can increase raw material dissolution and the development of geopolymer gels.

Accordingly, the concentration of activating solution sharply affects the compressive strength of SAP geopolymers. The low concentration of acid solution, approximately 2–4 mol L^−1^, showed the least compressive strength, whereas the samples obtained with acid solution concentrations of 6–14 mol L^−1^ showed good mechanical strength. This is because with low concentrations of acid solutions, there is a small number of PO_4_^3−^ ions that can bond aluminum from the raw material, which leads to the least formation of [AlPO_4_] tetrahedrals and thus low mechanical strength.

### 2.2. Geopolymerization mechanism of phosphate-geopolymers

**Fig. 8:**
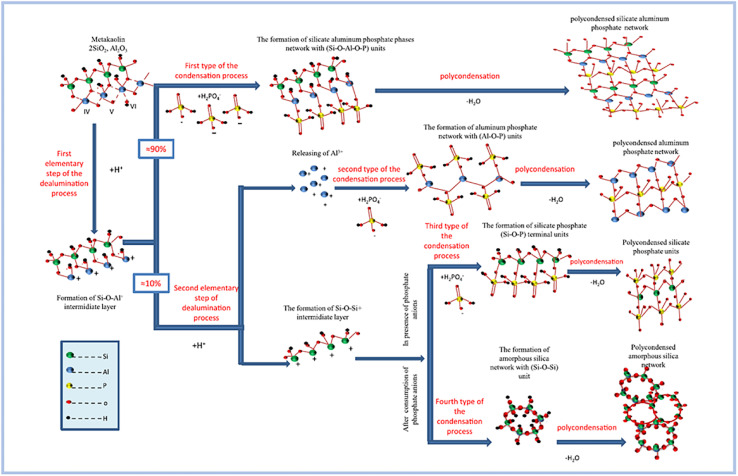
Conceptual model of the proposed formation mechanism of phosphate based geopolymers. This figure has been adapted/reproduced from ref. 56 with permission from Elsevier under the license number 5630020882161, copyright 2023.

Zribi and Baklouti,^52,56^ proposed the formation mechanism of SAP geopolymers, regarding which the geopolymerization process can be divided into three main steps and illustrated as follows (Fig. 8):

(1) The first stage is dealumination of the aluminosilicate precursor process. This phase is demonstrated to take place within the first 30 minutes of the examined synthesis setting. The procedure is further divided into two basic parts. The first assault attacks all Al–O–Al bonds, but the second is selective and only targets a subset of Si–O–Al bonds.

(2) As the second phase, condensation is carried out. This process is divided into four parts based on the chemical makeup of the reactant units and the resulting units: the synthesis of silicate aluminum phosphate phases, aluminum phosphate phases, silicate phosphate phases, and amorphous silica phases. This stage occurs at a varied rate from the beginning of the reaction until 12 hours have passed. In reality, as the reaction time increases, this pace reduces and becomes practically constant after 12 hours.

(3) The third stage is polycondensation. This stage begins during the first hour of the response and continues at a sluggish rate for several days. As a result, various 3D polymerized networks are formed.

## 3. Properties of phosphate-activated geopolymers and application

### 3.1. Thermal stability

Recent research has revealed that SAP geopolymers have high heat stability, which AAS geopolymers and ordinary Portland cement (OPC) do not have.

**Fig. 9:**
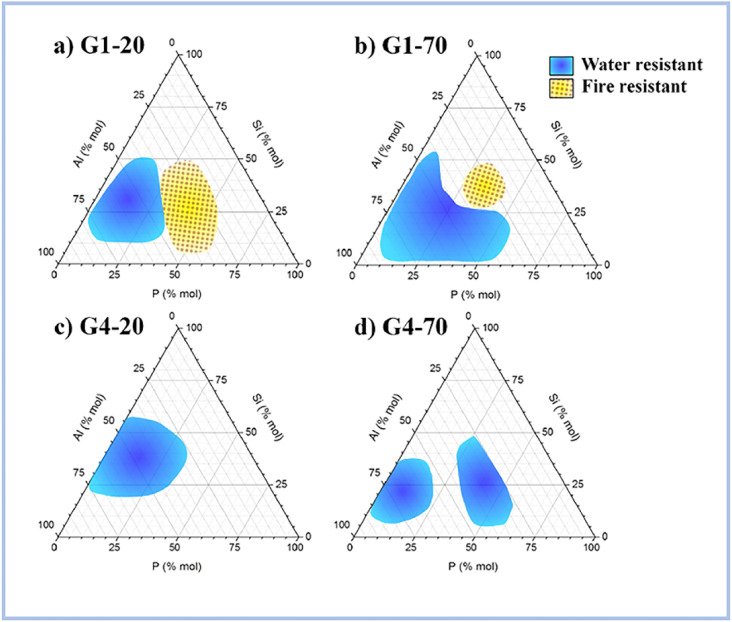
Compositions of the water-resistant (blue) and fire-resistant (yellow) geopolymers for (a) G1-20, (b) G1-70, (c) G4-20 and (d) G4-70. This figure has been adapted/reproduced from ref. 63 with permission from Elsevier under the license number 5630021267876, copyright 2023.

According to Liu *et al.* (2012),^62^ ASP geopolymers show excellent thermal stabilities at 1550 °C, whereas OPC would peel off or even rupture within at high temperatures. This is because geopolymer crystallizes at high temperatures to generate quarts, cristobalite, and aluminum phosphate, which provide exceptional thermal stability to geopolymer concretes. Celerier *et al.* (2018)^63^ discovered that the heat resistance of ASP geopolymers is dependent on the starting material, such as metakaolin, and its composition. Furthermore, the author stated testing at high temperatures several compositions that endured fire, implying that they did not become brittle following the treatment. Fig. 9 shows the compositions of water and fire-resistant geopolymers.

Thermal resistant geopolymer ceramics have potential uses such as furnace refractory boards, casting mold cores, fire-retardant concrete, runway pavement, and so on. These applications necessitate strong thermal and mechanical qualities. These applications need strong temperature resistance as well as adequate mechanical qualities. However, conventional geopolymer materials lack mechanical strength as well as heat resistance. Fiber reinforcing is recognized as an effective approach for improving ceramics' mechanical and thermal resistance. To improve the characteristics of geopolymers, a variety of fibers are used as reinforcing phases, including natural fibers such as cotton fiber,^64^ wood fiber,^65^ hemp fiber,^66^ and others.^67,68^ Ceramic fibers, such as silicon carbide fibers, have also been studied as reinforcing phases.^69,70^ Fang *et al.* (2021)^71^ examined the temperature-dependent property evolution of geopolymer composites with BN-coated SiC whiskers. Up to 900 °C, the composites demonstrated enhanced high-temperature strength.

**Fig. 10:**
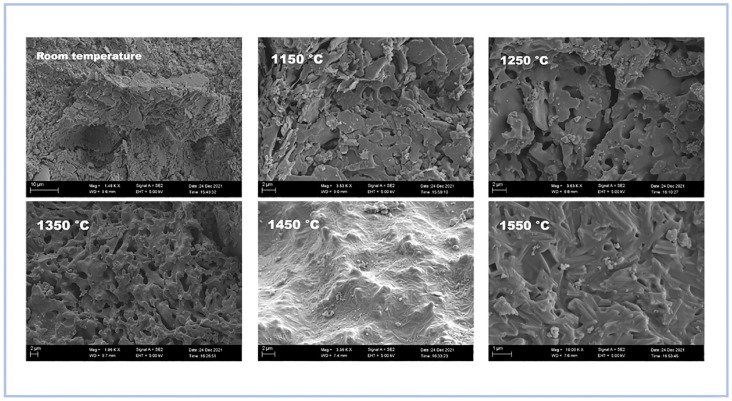
SEM images of samples treated at different temperatures, Wei *et al.*, *Materials*, 2022.^72^

Wei *et al.* (2022)^72^ examined the thermal characteristics of SAP modified with mullite fibers and discovered that mullite fiber has a high affinity for phosphate geopolymer and has a considerable fiber reinforcing effect. As shown in the Fig. 10, despite the presence of microcracks on the surface of the obtained samples, modified geopolymers demonstrated remarkable thermal stability at 1350 °C, however at temperatures over 1450 °C, geopolymers began to breakdown owing to AlPO_4_ melting.

In consequence, SAP geopolymers have gained a potential number of applications in the heat and fire resistance materials area due to their high thermal stability. This property depends on the mineralogical constitution of the raw materials used in the preparation process and the transformation of minerals under high-temperature treatment. Moreover, the reinforcement of SAP geopolymers with mullite fibers and silicon carbide fibers also increases the heat resistance of geopolymer concrete.

### 3.2. Thermal insulation

Thermal insulation is defined as the decrease of heat transfer between objects in thermal contact or within the range of controlled radiative.^73^ Thermal insulating material can be defined as a single substance or mixture of materials that, when appropriately applied, slows the rate of heat transport by conduction, convection, and radiation. Because of its high thermal resistance, it slows heat movement into or out of an object or structure.^74^ Thermal insulation can be done by the use of properly designed technologies or processes, as well as appropriate object forms and materials. Thermal insulation creates an insulating area in which thermal conduction is decreased or thermal radiation is reflected rather than absorbed by the lower-temperature substance.^75^

Thermal insulating materials impede heat flow as a result of innumerable tiny dead air-cells that inhibit convective heat transmission. The thermal resistance is provided by the air trapped within the insulation, not by the insulation material itself. Making holes or tiny cells within thermal insulation with a slight temperature differential decreases radiation impact. It breaks down radiation “paths” into short lengths where long-wave infrared radiation is absorbed and dispersed by the insulating material.^74^

Inorganic materials (glass, rock, slag wool, ceramic goods, geopolymers) and organic materials (cellulose, cotton, wood, foamed rubber, polystyrene, polyethylene, polyurethane) are examples of acceptable thermal insulation materials. However, geopolymers have been used to create more effective insulating materials. SAP geopolymers have been studied as thermal insulation materials by several researchers and have received a lot of interest due to their remarkable heat insulating capabilities.

**Fig. 11:**
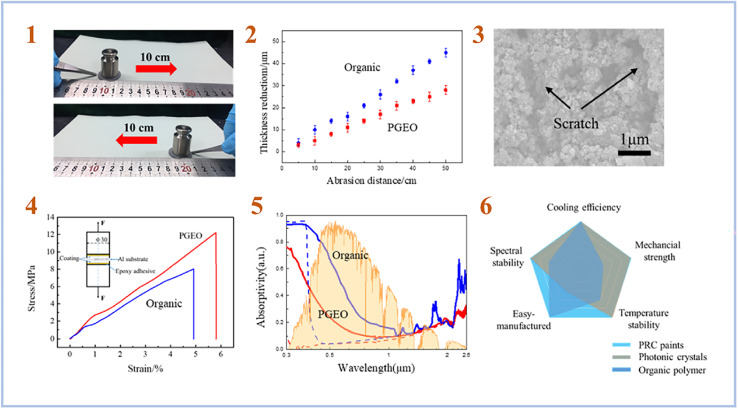
(1) Photographs showing the mechanical linear abrasion test performed on sandpaper (standard glasspaper, grit no. 240) with 100 g of abrading load; (2) comparison of the thickness reduction of the PGEO and the organic DRC coatings at different linear abrasion distances; (3) SEM surface morphology of the coating after an abrasion distance of 50 cm; (4) comparison of the adhesive strength of the PGEO coating and the organic DRC coating to an Al substrate; (5) measured solar spectral reflectance of the PGEO coating compared to that of the organic DRC coating before (dashed lines) and after (solid lines) space proton irradiation; (6) overall comparison of performance for the as-prepared PGEO coating, photonic crystals, and organic polymer radiative cooling designs. This figure has been adapted/reproduced from ref. 76 with permission from American Chemical Society, copyright 2023.

Chen *et al.* (2020)^76^ created a Daytime Radiative Cooling (DRC) coating using SAP geopolymers. In order to create a strong phosphate geopolymer (PGEO) covering, they employed metakaolinite as a starting material and a mixture of phosphoric acid and aluminum hydroxide as an activating solution. In the solar irradiation band, the produced PGEO covering with a thickness of 50 μm exhibits almost 90% reflectance. Under direct sunlight, the coating's exceptional spectrum selectivity results in a subambient temperature reduction at the coated surface of up to 8.3 °C. Furthermore, the spectrum selectivity of the PGEO coating was maintained across a variety of extreme environmental conditions, including high temperature, mechanical abrasion, and proton irradiation (Fig. 11). As a result, this PGEO covering has prospective applications in solar-exposed components such as spacecraft and buildings.

Gualtieri *et al.* (2015)^77^ created foamed SAP geopolymers with limestone as the pore foaming agent and metakaolin. The geopolymers obtained have a high total porosity (68–70%) and a low effective thermal conductivity (0.092–0.095 W m^−1^ K^−1^). Morsy *et al.* (2019)^78^ did a similar study and found that SAP geopolymers can be employed as heat insulating materials due to their low thermal conductivity (0.211 W m^−1^ K^−1^). They also studied if it is feasible to employ SAP geopolymers without the use of a foaming agent, because thermal conductivity data were achieved with non-foamed SAP geopolymer samples.

**Fig. 12:**
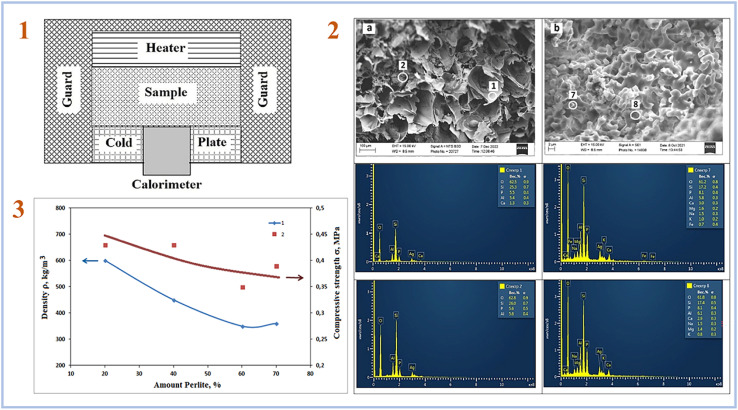
(1) Scheme of facility for measuring thermal conductivity; (2) the structure of the cleavage of composite No. (1) Fired at 1000 °C a – filler and b – binder; (3) effect of filler content on the properties of composite ceramics based on perlite, heat-treated at 1000 °C; (1) density. (2) Compressive strength. This figure has been adapted/reproduced from ref. 79 with permission from Elsevier under the license number 5630030100032, copyright 2023.

Rumi *et al.* (2023)^79^ employed perlite-expanded clay to manufacture SAP geopolymers and 20–40 wt% phosphoric acid solutions as an acid-based activator in their latest study. As a result, it has been demonstrated that the inclusion of the specified amounts of phosphoric acid allows the production of heat-insulating materials based on perlite-clay SAP geopolymers. The thermal conductivity of the tested samples ranged from 0.09 to 0.43 W m^−1^ K^−1^. Fig. 12 depicts a facility for assessing thermal conductivity, SEM pictures of samples, and compressive strength of samples.

Rashad *et al.* (2023)^88^ manufactured SAP geopolymers from metakaolin using sugar beet waste as a foaming agent and investigated the thermal insulation capabilities of the resulting samples. As a result of applying 6% foaming agent, the lowest thermal conductivity of the produced geopolymers was 0.08 W m^−1^ K^−1^. This value is significantly lower than that of other alkaline-activated insulating materials. They also evaluated the thermal conductivity of typical insulating materials and found that geopolymers foamed with sugar beet waste may be employed in large-scale thermal insulation applications.

Jouin *et al.* (2023)^89^ created SAP geopolymers out of metakaolin and compared their thermal conductivity with the properties of other insulating materials. They reviewed the literature and determined that SAP geopolymers may be utilized in the building of insulating applications, as well as in the field of refractories to lighten bricks and for filtering.

Hence, SAP geopolymers have least thermal and radioactive conductivity due to their morphology and porous structure. Regarding the mentioned research, SAP geopolymers have found their application in different areas, for instance, as daytime radiative cooling coatings in spacecraft and construction and as excellent heat insulating materials widely used in construction.

### 3.3. Porosity and porous foam materials

At the present time, the production of SAP geopolymer porous foam materials by adding foaming agents is a sufficient direction in the SAP geopolymer application study. SAP geopolymer porous foam materials are not only heat and fire resistant but also light weight, heat insulation, and have a high sorption capacity, making them a possible novel type of thermal insulation material and adsorbents.^62^ Aluminum powder,^60,80^ iron powder,^80^ surfactant,^81^ hydrogen peroxide,^82^ and limestone^77,78^ are now applied as foaming agents.

The fundamental concepts for creating SAP geopolymer foams may be separated into two groups. One method is to apply foaming agents such as aluminum/iron powder or limestone to react with alkali or acid to create gas, which results in a homogeneous and rich foam structure when the geopolymer paste is formed. The alternative option is to employ a surfactant-based foaming agent that is directly combined with the slurry.

Porous geopolymers may be produced from metakaolin and H_3_PO_4_ using aluminum powder as a foaming agent, according to Le-ping *et al.* (2010).^60^ The porosity of the foamed geopolymer samples ranged from 40% to 83%, with compressive strength ranging from 7 to 13 MPa.

Shuai *et al.* (2019)^82^ created SAP foamed geopolymers from metakaolin and foamed them with hydrogen peroxide solutions at concentrations of 2, 2.5, 3, 3.5, and 4%. The porosity of the produced samples ranged from 55% to 64%, with the greatest compressive strength achieved at 4% and 2% H_2_O_2_ solutions.

Bai *et al.* (2019)^81^ used the surfactant Triton X-100 as a foaming agent in the preparation process of SAP geopolymer foam material from metakaolin. The obtained geopolymers showed high mechanical strength, fire resistance, and porosity. They reported that geopolymers foamed with surfactant are eco-friendly and can be used in construction.

Morsy *et al.* (2019)^78^ combined metakaolin with limestone to produce foamed SAP geopolymers. As a result of their research, they obtained highly porous geopolymers with low thermal conductivity and reasonable mechanical strength.

**Fig. 13:**
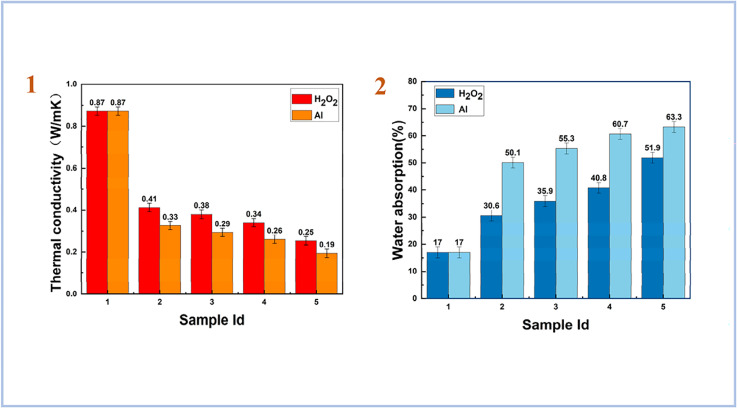
(1) thermal conductivity of HMNS-FA-phosphate-based porous geopolymers; (2) water absorption (%) of HMNS-FA-phosphate-based porous geopolymers, Yang *et al.*, *Minerals*, 2023.^83^

Yang *et al.* (2023)^83^ used both aluminum powder and hydrogen peroxide as foaming agents with a combination of high magnesium nickel slag (HMNS) and fly ash (FA) as starting material. As a result, they have obtained porous materials with a low dry density and low thermal conductivity. For geopolymers foamed with H_2_O_2_, the results of thermal conductivity were higher than for samples foamed with Al. However, for water absorption tests, samples foamed with Al showed the highest result compared to samples foamed with H_2_O_2_ (Fig. 13). They also concluded that HMNS-FA foamed SAP geopolymers can be used as thermal insulation materials and as water storage materials.

Rashad *et al.* (2023)^88^ employed carbonated lime residue (CR), a waste product of the sugar beet production sector, as a foaming agent in the synthesis of metakaolin-based porous geopolymers. As a consequence, they concluded that CR can be used as a foaming agent since the foamed geopolymers generated had low heat conductivity and reasonable compressive strength. The collected samples have a total porosity of 61.2% and a bulk density of 631.5 kg m^−3^.

According to the mentioned studies, SAP geopolymers can be easily foamed with different types of foaming agents, starting with aluminum powder and ending with surfactants. Moreover, foamed SAP geopolymers have great mechanical properties, the lowest thermal conductivity, and high adsorption properties, which gives them a wide range of applications in different areas.

### 3.4. Adsorption properties

Adsorption is recognized as one of the most successful wastewater treatment strategies due to the availability of a variety of adsorbent materials that are inexpensive in cost, readily available, and can be manufactured utilizing a simple step of synthesis.

Previous research has demonstrated that SAP geopolymers have good adsorption and solidification properties for heavy metals and radioactive nuclear waste.^84^ Phosphate-based geopolymers can effectively prevent heavy metal ion or radioactive element leaching by firmly blocking heavy metal ions in the cavity of their unique three-dimensional network structure.

Khan *et al.* (2015)^85^ investigated phosphate-based geopolymers for wastewater treatment by removing methylene blue (MB) from water using SAP geopolymers. As a result, SAP geopolymers easily absorbed MB from water, according to their findings. Furthermore, geopolymers are thermally stable and may be regenerated several times without compromising adsorption capabilities.

Njimou *et al.*^86^ investigated the removal of Pb(ii) ions from aqueous solutions using a SAP geopolymer–alginate composite. The resulting SAP geopolymer–alginate composite (Alg/CES) beads had an adsorption capacity of 0.183 mmol g^−1^, a pH of 4.27, and an initial concentration of 113.4 mg L^−1^. They determined that such geopolymer composite is environmentally benign and suited for heavy metals wastewater treatment.

**Fig. 14:**
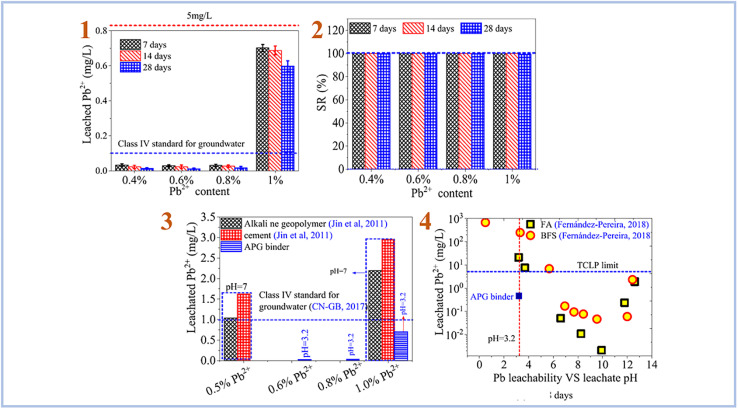
(1) leaching rate of Pb^2+^ in SAP geopolymer binder; (2) retention rate of Pb^2+^ in SAP geopolymer binder; (3) comparison of the stabilization effects of SAP (APG) binder, cement and alkali-activated geopolymer on Pb^2+^; (4) comparison of the stabilization effects of SAP (APG) binder, cement and alkali-activated geopolymer based on FA and BFS on Pb^2+^. This figure has been adapted/reproduced from ref. 87 with permission from Elsevier under the license number 5630030293444, copyright 2023.

Pu *et al.* (2021)^87^ investigated Pb^2+^ ion stabilization in SAP geopolymer binder (Fig. 14). They discovered that SAP geopolymers have a strong stabilizing effect on Pb^2+^ and outperform alkali-activated geopolymer and cement.

**Fig. 15:**
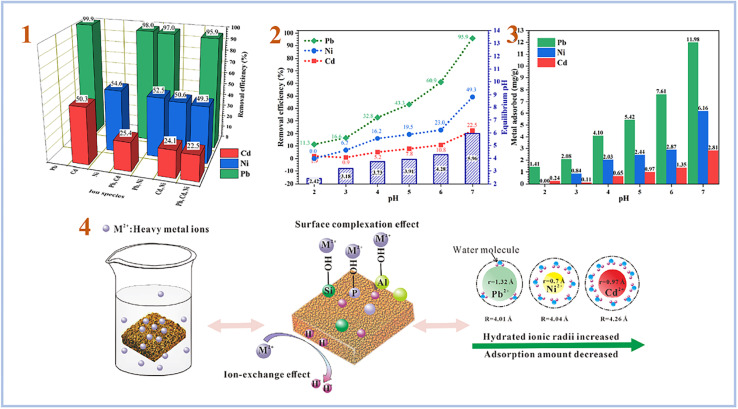
(1) Removal efficiency of Pb, Ni and Cd by PAG (SAP) from single, binary and ternary mixtures; (2) effect of pH on the removal efficiency, equilibrium pH; (3) metal adsorption amount of Pb, Ni and Cd by PAG (SAP) in ternary mixtures; (4) possible mechanism of heavy metals adsorption by phosphoric acid-based geopolymer. This figure has been adapted/reproduced from ref. 90 with permission from Elsevier under the license number 5630030483481, copyright 2023.

Liu *et al.* (2022)^90^ tested the adsorption of combinations of Pb(ii), Cd(ii), and Ni(ii) using foamed SAP geopolymer. Metakaolin was used to create geopolymers, which were then foamed with H_2_O_2_ and stabilized with Triton X-100. As a consequence, they discovered that the pH of the solution plays an important role in heavy metal adsorption, with pH 7 providing the best adsorption effectiveness for heavy metal ions (Fig. 15). Furthermore, they compared the sorption performance of SAP geopolymers to that of AAS geopolymers and found that the sorption performance of SAP geopolymers is much greater than that of AAS geopolymers.

Therefore, SAP geopolymers have gained huge attention in wastewater treatment applications because of their microstructure and morphology. Geopolymers are able to immobilize heavy metal ions inside their three-dimensional network structure, which is necessary in current ecological conditions. SAP geopolymers can be widely used in production due to the fact that their results on sorption capacity and leaching tests exceed those of AAS geopolymers.

### 3.5. Durability

One of the most crucial characteristics required in the development of geopolymer applications is durability. Water resistance is the most important feature in geopolymer engineering application index.

According to Mimboe *et al.* (2020),^91^ the strength loss of SAP geopolymer based on laterite soaking in water for 24 h surpasses 60% of dry compressive strength. Ndjock study (2021)^92^ discovered that SAP geopolymers immersed in water for 24 hours had a 54% loss in compressive strength, whereas Nobouassia *et al.* (2018)^93^ discovered a 54% decrease in compressive strength after soaking in water for 28 days.

However, the endurance of SAP geopolymers can be enhanced by adding a Ca source, such as slags. According to Djobo and Stephan *et al.* (2021),^94^ geopolymers made with pozzolanic ash lowered the strength loss rate from 40.3% to 24.7%. They also discovered that after soaking SAP geopolymer samples in water, the binder began to breakdown, resulting in white powder on the surface of the binder.^95^ Kaze *et al.* (2021)^96^ created SAP geopolymer binder using iron-rich laterite. As a consequence, they determined that the iron oxide composition of SAP geopolymers boosts durability and creates stronger water resistance than AlP geopolymers.

**Fig. 16:**
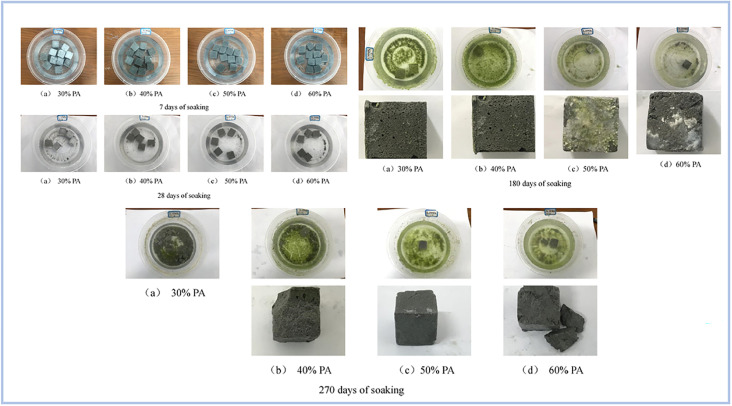
Evolution of geopolymer specimens different soaking times, Pu *et al.*, *Dev. Built Environ.*, 2022.^97^

Pu *et al.* (2022)^97^ investigated the water resistance capabilities of produced binders using FA as the raw material for SAP geopolymer samples. Samples were soaked for 7, 28, 180, and 270 days. As a consequence, samples submerged for 7 and 28 days lost 40% of their strength, whereas samples soaked for 180 days had no visible fractures on their surface. However, after soaking for 280 days, the samples grew fragile and began to break (Fig. 16).

Therefore, the main issue with SAP geopolymers in their current state is durability. According to previous studies, SAP geopolymer binders have a decrease in compressive strength of 40–60%. However, depending on the constitution of the raw material used for the preparation process of SAP geopolymers, the loss of compressive strength can be decreased. However, this area of study needs to be studied more because durability is one of the major factors in the production of construction materials.

## 4. Possible applications in the future

According to the investigations, SAP geopolymers have numerous beneficial characteristics, including strong compressive strength,^51^ good heat resistance,^71,72^ and high sorption activity.^85,86,90^ However, there are certain impending concerns with SAP geopolymers, such as a long polycondensation time at room temperature, and low water and corrosion resistance. As a result, we can forecast the potential application direction of SAP geopolymers in the future based on their strengths and negative aspects.

SAP geopolymers' outstanding mechanical qualities and heat resistance, when compared to other commonly used construction materials, allow them to be employed in construction and building materials. However, the fact that SAP geopolymers can solidify quicker only at increased temperatures, about 60–80 °C, might be mentioned as a major limitation in SAP geopolymer manufacture. As a result, raising the temperature during binder curing increases expenses as well as CO_2_ gas pollution. A further weakness of SAP geopolymers is their low water endurance, which leads to material failure. As an outcome of all the research reviewed, there are just two main drawbacks that merit further investigation. For example, the endurance of SAP geopolymers can be extended by adding certain components to the binder or by employing starting materials with high calcium or iron oxide constitutions, which give greater water resistance. As a result, all problems in SAP geopolymers may be overcome, and there are many more benefits than we can anticipate.

A prior investigation has shown that SAP geopolymers may be formed as foams featuring good heat resistance, low thermal conductivity, and good water adsorption, and can be used as a lightweight insulation material.^83^ Furthermore, SAP geopolymers with high hardness, spectrum selectivity, and adhesion can be used as daytime radiative cooling coatings in urban construction and spacecraft.^76^ Furthermore, SAP geopolymers have strong heavy metal adsorption characteristics, making them promising materials for wastewater treatment.^85,86,90^

From all aforementioned, despite minor difficulties that may be resolved, we believe SAP geopolymers have a bright future in the construction industry.

## 5. Conclusion

This study analyzes the most recent advances in silico-aluminophosphate (SAP) geopolymer production, categorization, characteristics, and applications. SAP geopolymers have superior mechanical qualities, high heat and fire resistance, low thermal conductivity, and strong sorption activity, according to the present research. These features make SAP geopolymers green building materials with a wide range of applications in insulating materials, coating materials, and wastewater treatment. Thoroughly minimizing the issue of curing conditions and enhancing the durability of SAP geopolymers would aid in the promotion of SAP geopolymer industrialization applications.

## Conflicts of interest

There are no conflicts to declare.

## Supplementary Material
